# Platelets, gender and acute cerebral infarction

**DOI:** 10.1186/s12967-015-0630-x

**Published:** 2015-08-16

**Authors:** Petter Järemo, Marie Eriksson-Franzen, Micha Milovanovic

**Affiliations:** Department of Internal Medicine, The Vrinnevi Hospital, 601 82 Norrköping, Sweden

**Keywords:** Flow cytometry, Gender, Inflammation, Myeloperoxidase, Platelet activation, Platelet reactivity, Platelets, P-selectin

## Abstract

**Objective:**

Platelets may well be significant in the pathogenesis of cerebral infarction. Platelets vary substantially according to gender. The scope of our current work is to establish if female and male stroke sufferers differ regarding platelet reactivity.

**Patients and methods:**

73 Consecutive individuals stricken by acute ischemic cerebral infarction (31 females, 42 males) participated. All stroke subtypes were included. Platelet counts was determined electronically. Platelet reactivity i.e. the presence of surface-bound fibrinogen following provocation was analyzed with a flow cytometer. ADP (1.7 μmol/L) and a thrombin receptor agonist (TRAP-6) (57 μmol/L) were the agonists used.

**Results:**

Female stroke sufferers had higher platelet counts (*p* = 0.013) but their platelets were less reactive. The *p* values were (*p* = 0.038) and (*p* = 0.016) for ADP and TRAP-6, respectively.

**Conclusion:**

The current study demonstrates that women suffering acute cerebral infarction have less reactive platelets. It is concluded that gender affects platelets. Our study indicates that it may be beneficial to individualize platelet inhibition of stroke sufferers according to gender.

## Background

Drugs that suppress platelets e.g. aspirin, are beneficial for preventing stroke recurrence [1]. Some workers have associated acute cerebral infarctions with increased platelet reactivity [2]. In contrast, recent work have found either lower platelet reactivity [3] or no reactivity alterations [4]. Research into platelet activity in the event of acute stroke has also produced disparate findings. Several researchers found increased platelet activity [5, 6] whereas recent work has revealed reduced activation [3, 4]. Platelets display substantial gender differences; women have increased platelet counts [7, 8] and female platelets display increased sensitivity to aggregating agents both before [8, 9] and after [7, 10] the menopause. The diversities encouraged us to undertake the current work which evaluates stroke victims with respect to gender differences in platelet behavior.

## Methods

### Study subjects

We enrolled individuals (*n* = 73) stricken by acute cerebral infarction admitted to a specialized stroke unit. The condition was defined as an abrupt loss of neurological functions exceeding 24 h. Brain hemorrhage cases and unconscious patients were excluded. Otherwise, no exclusion criteria were applied. We divided the disease into subtypes; viz. cardioembolism (*n* = 14), small artery disease (*n* = 38) and major stroke (*n* = 21). Subsequently, the subjects were divided according to gender (31 women, 42 men) (Table 1). The study was approved by the Regional Ethical Review Board (Linköping, Sweden).

**Table 1:** Demographic data at hospital admission for females and males with acute cerebral infarctions

	Females	Males	*p* value
Subjects (*n*)	31	42	
Age (years)^a^	78 ± 9	71 ± 9	0.001
Body weight (kg)^a^	70 ± 16	83 ± 13	0.004
Sampling time after the acute stroke (days)^a^	2.4 ± 1.6	2.0 ± 1.3	NS
Cardiogenic stroke (*n*)	8	6	NS
Small artery disease (*n*)	14	24	NS
Large artery disease (*n*)	9	12	NS
Recurrent stroke (*n*)	6	15	NS
Current smokers (*n*)	3	7	NS
Diabetes (*n*)	2	9	NS
Hypertension (*n*)	11	21	NS
Previous myocardial infarction (*n*)	2	5	NS
A_2_-Blockers (*n*)	1	3	NS
ACE-inhibitors (*n*)	13	4	0.001
Aspirin 75 mg (*n*)	11	19	NS
β-Blockers (*n*)	10	11	NS
Ca^2+^-Blockers (*n*)	6	9	NS
Clopidogrel *(n)*	0	0	NS
Diuretics (*n*)	5	9	NS
Statins (*n*)	4	11	NS
Vitamin K antagonists (*n*)	1	5	NS

*NS* not significant.

^a^mean ± SD.

### Analytical procedures

Determinations of surface-bound fibrinogen and membrane-attached P-selectin were carried out in citrate anticoagulated whole blood with a Beckman Coulter EPICS XL-MCL™ Flow Cytometer (Beckman Coulter, Inc., Brea, Cal, USA). Earlier communications have described the laboratory procedures in detail [4, 7, 11]. An antibody against glycoprotein Ib (Dako AS, Glostrup, Denmark) detected platelets. Chicken antihuman fibrinogen polyclonal antibody (Biopool AB, Umeå, Sweden) identified surface-bound fibrinogen. An IgG1 (mouse) monoclonal antibody identified platelet bound P-selectin (Immunotech, France). The values of a negative control were subtracted from the experimental ones. The control contained EDTA to prevent platelet antibody binding. When determining platelet reactivity, ADP (1.7 and 8.5 μmol/L) (Sigma-Aldrich, St Louis, MO, USA) and a thrombin receptor-activating peptide-6 (TRAP-6) (57 and 74 μmol/L) (Biotechnology Centre of Oslo, Norway) were used as agonists. Platelet-bound P-selectin without agonist provocation served as an estimate of platelet activity in vivo [4, 11]. Soluble P-selectin and myeloperoxidase were used as markers of platelet/endothelial [11, 12] and neutrophil activity [13], respectively. ELISA kits (R&D system, Abingdon, GB) were employed for both determinations. To avoid platelet in vitro activity, a blocking solution was used as an anticoagulant [4, 14]. High sensitive C-reactive protein (hsCRP) was determined using a turbiometric technique. Student’s *t* test and the Chi square test were employed for the statistical evaluations.

## Results

### Demographic data

Female stroke sufferers were older than their male counterparts (Table 1). As expected female body weights were lower. At hospital admittance neither stroke subtypes nor concomitant diseases differed significantly with respect to gender. With one exception (ACE-inhibitors) the study groups had similar drug prescriptions.

### Platelet reactivity and activity

At the acute stroke, female participants had increased platelet counts (Table 2) and their platelet distribution width was narrower. Platelet reactivity as estimated from platelet fibrinogen binding following agonist stimulation varied such that females had less reactive platelets. Provocation with more concentrated ADP and TRAP-6 also revealed a tendency towards lower platelet reactivity in females though the differences failed to reach statistical significance (Table 2). Platelet reactivity, as estimated from membrane-bound P-selectin after TRAP-6 stimulation, proved to be unrelated to gender. Finally, platelet activity, as estimated from surface-attached P-selectin without agonist provocation and from circulating P-selectin, was not associated with sexual category (Table 2).

**Table 2:** Platelet reactivity and activity of female and male stroke sufferers

	Females (mean ± SD) (*n* = 31)	Males (mean ± SD) (*n* = 42)	*p* value
Platelet counts (×10^9^/L)	295 ± 105	245 ± 58	0.013
Platelet distribution width (%)	15.7 ± 1.7	16.4 ± 0.9	0.03
Platelet-bound fibrinogen after agonist provocation			
ADP (1.7 μmol/L) (%)	23 ± 16	32 ± 17	0.038
ADP (8.5 μmol/L) (%)	54 ± 21	62 ± 19	NS
TRAP-6 (57 μmol/L) (%)	35 ± 23	52 ± 29	0.016
TRAP-6 (74 μmol/L) (%)	51 ± 24	63 ± 22	NS
Platelet-bound P-selectin after agonist provocation			
TRAP-6 (57 μmol/L) (%)	20 ± 13	15 ± 7	NS
TRAP-6 (74 μmol/L) (%)	41 ± 22	44 ± 18	NS
Platelet-bound P-selectin			
Without agonist (%)	4 ± 2	4 ± 1	NS
Soluble P-selectin (μg/L)	70 ± 37	82 ± 47	NS

*NS* not significant, *%* percentage positive cells (either fibrinogen or P-selectin), *TRAP-6* thrombin receptor activating peptide.

### Inflammatory response and erythrocytes

Females displayed augmented inflammatory response judging from neutrophil counts (Table 3). At the acute stroke, their neutrophils showed increased activity as estimated from plasma myeloperoxidase. In contrast, hsCRP showed no gender differences. Female stroke sufferers then had lower hemoglobin concentrations and increased red cell distribution width whereas their erythrocyte counts were similar to those of male controls (Table 3).

**Table 3:** The inflammatory response and red cell parameters for female and male patients with acute cerebral infarctions

	Females (mean ± SD) (*n* = 31)	Males (mean ± SD) (*n* = 42)	*p* value
High sensitive C-reactive protein (g/L)	17 ± 18	9 ± 17	NS
Neutrophil counts (×10^12^/L)	5.6 ± 1.7	4.6 ± 1.6	0.011
Myeloperoxidase (μg/L)	16 ± 13	7 ± 6	0.006
Hemoglobin (g/L)	141 ± 13	149 ± 13	0.019
Red cell counts (×10^9^/L)	4.6 ± 0.5	4.8 ± 0.5	NS
Red cell distribution width (%)	13.2 ± 1.4	12.4 ± 0.9	0.007

*NS* not significant.

## Discussion

This work has revealed gender differences concurrent with acute stroke. Female stroke sufferers displayed less platelet reactivity (Table 2). We further confirm earlier findings [7] in showing that females had higher platelet counts. Finally, at the acute stroke women had enhanced neutrophil counts. These cells circulate more activated in females based on plasma myeloperoxidase values (Table 3).

We found that platelets of women stroke patients responded less to agonist (Table 2). Previous work showed increased female platelet reactivity concurrent with coronary heart disease [7]. Furthermore, women with atherosclerosis have increased reactivity [10]. Several researchers describe how platelets of stroke sufferers are less reactive than in suitable control groups [3, 4]. It is difficult to decide why coronary heart disease platelets differ from platelet behavior in conjunction with cerebral infarction. One can hypothesize that stroke and coronary heart disease have differing pathologies making platelets react differently.

Stroke incidence is lower in women and they are on average older than males at their first cerebral infarction [15–17]. Female stroke patients have higher prevalence of hypertension and cardioembolic stroke whereas diabetes and lacunar stroke are less frequent [17]. It agrees to a certain extent with this study. In our hands, however, probably due to the small sample size, the differences failed to reach significance (Table 1). Demographic and clinical characteristics differed further as women weighted less and more frequently had ACE-inhibitors (Table 1). In particular, differences with respect to ongoing prescriptions could affect platelets and inflammatory reactions. The time point of blood sampling varied substantially but did not differ significantly between groups (Table 1). It constitutes an uncertainty as the current activity measures could rapidly change after an acute cerebral infarction. Female patients were older at hospital admission constituting a possible confounder. However, in our hands platelets and neutrophils do not demonstrate any significant dependence on age (unpublished data). In everyday clinical practice it is practically impossible to divide stroke events into subcategories. Previously, we were unable to see that stroke subtypes differ with respect to platelets and the inflammatory response [4]. Therefore, in this study all patients were evaluated together.

Lower platelet reactivity could be one of many reasons as to why women on average get their first cerebral infarction later in life. Female stroke sufferers have higher 1-month case fatality than men [15]. Age and disease severity may be possible reasons for the dissimilarities [16, 17]. The inflammatory response has substantial impact upon long term survival after coronary heart disease [18]. It is to evaluate if inflammatory parameters may have prognostic influence after an acute cerebral infarction as well.

## Conclusion

The current study indicates that subsequent work is necessary to evaluate if platelet inhibition after acute stroke should be individualized according to gender. At present new powerful platelet inhibitory drugs are introduced. This work suggests that clinical trials are necessary before using these new drugs in particular in female stroke sufferers.
